# Healthcare provider characteristics that influence the implementation of individual-level patient-centered outcome measure (PROM) and patient-reported experience measure (PREM) data across practice settings: a protocol for a mixed methods systematic review with a narrative synthesis

**DOI:** 10.1186/s13643-021-01725-2

**Published:** 2021-06-09

**Authors:** Angela C. Wolff, Andrea Dresselhuis, Samar Hejazi, Duncan Dixon, Deborah Gibson, A. Fuchsia Howard, Sarah Liva, Barbara Astle, Sheryl Reimer-Kirkham, Vanessa K. Noonan, Lisa Edwards

**Affiliations:** 1 School of Nursing, Trinity Western University, 22500 University Drive, Langley, British Columbia V2Y 1Y1 Canada; 2 Department of Evaluation and Research Services, Fraser Health Authority, Suite 400, 13450 – 102nd Avenue, Surrey, BC V3T 0H1 Canada; 3 N.M. Alloway Library, Trinity Western University, 22500 University Drive, Langley, BC V2Y 1Y1 Canada; 4 Faculty of Applied Sciences, School of Nursing, The University of British Columbia, Vancouver Campus, T201 - 2211 Wesbrook Mall, Vancouver, British Columbia V6T 2B5 Canada; 5 Research and Best Practice Implementation, Praxis Spinal Cord Institute, 818 West 10th Avenue, Vancouver, BC V5Z 1M9 Canada; 6 Faculty of Health Studies, University of Bradford, Richmond Road, Bradford, West Yorkshire BD7 1DP UK

**Keywords:** Patient-reported outcome measures (PROMs), Patient-reported outcomes (PROs), Routine outcome monitoring (ROM), Patient-reported experience measures (PREMs), Knowledge translation (KT), Patient-centered care (PCC), Clinical decision-making, Clinicians, Healthcare providers, Implementation

## Abstract

**Background:**

Substantial literature has highlighted the importance of patient-reported outcome and experience measures (PROMs and PREMs, respectively) to collect clinically relevant information to better understand and address what matters to patients. The purpose of this systematic review is to synthesize the evidence about how healthcare providers implement individual-level PROMs and PREMs data into daily practice.

**Methods:**

This mixed methods systematic review protocol describes the design of our synthesis of the peer-reviewed research evidence (i.e., qualitative, quantitative, and mixed methods), systematic reviews, organizational implementation projects, expert opinion, and grey literature. Keyword synonyms for “PROMs,” PREMs,” and “implementation” will be used to search eight databases (i.e., MEDLINE, CINAHL, PsycINFO, Web of Science, Embase, SPORTDiscus, Evidence-based Medicine Reviews, and ProQuest (Dissertation and Theses)) with limiters of English from 2009 onwards. Study selection criteria include implementation at the point-of-care by healthcare providers in any practice setting. Eligible studies will be critically appraised using validated tools (e.g., Joanna Briggs Institute). Guided by the review questions, data extraction and synthesis will occur simultaneously to identify biographical information and methodological characteristics as well as classify study findings related to implementation processes and strategies. As part of the narrative synthesis approach, two frameworks will be utilized: (a) Consolidated Framework for Implementation Research (CFIR) to identify influential factors of PROMs and PREMs implementation and (b) Expert Recommendations for Implementing Change (ERIC) to illicit strategies. Data management will be undertaken using NVivo 12^TM^.

**Discussion:**

Data from PROMs and PREMs are critical to adopt a person-centered approach to healthcare. Findings from this review will guide subsequent phases of a larger project that includes interviews and a consensus-building forum with end users to create guidelines for implementing PROMs and PREMs at the point of care.

**Systematic review registration:**

PROSPERO CRD42020182904.

**Supplementary Information:**

The online version contains supplementary material available at 10.1186/s13643-021-01725-2.

## Background

Shifting from traditional, biomedical, disease-focused, and scientific models of healthcare toward patients being drivers of their care is essential to improve health and clinical outcomes [1]. Person-centered care that focuses on what matters to patients occurs when healthcare providers (HCPs) include and listen to the voice of patients. To do this, data collected from patient-centered measurement (PCM) can be used by HCPs to incorporate patients’ voice and inform their care [2]. PCM is an umbrella term used in this systematic review to include tools for measuring patients’ experiences or outcomes [3]. Substantial literature has highlighted the importance of PCM to collect clinically relevant information from patients to better understand and address what matters to them. Patient-reported outcome measures (PROMs) are questionnaires (also referred to as assessment tools or instruments) to measure patients’ health status in a standardized and quantitative manner at a point in time [3]. These inquiries may capture how patients function or feel with respect to their health; disease condition and its treatment; or functional status, quality of life, or mental well-being [4, 5]. In contrast, patient-reported experience measures (PREMs) focus on how patients feel with respect to their healthcare or illness experience. PREMs are commonly used to solicit information about patients' satisfaction with service delivery in a clinical setting, or describe patient experience of a therapy or plan of care [3]. To inform and plan patient care, PROMs and PREMs can be collected by HCPs for various purposes: screening, assessment, monitoring, planning, and evaluating care (e.g., interventions, treatment, referrals, and tests) as well as creating decision aids [6, 7]. Both PROMs and PREMs are intended to provide assessment data about patients’ health thereby complimenting HCPs use of clinician-based outcomes (CBOs), biological measures, and physical examination. When developed with input from patients and regularly integrated into clinical practice, PROMs and PREMs data can encourage conversations between patients and HCPs. As a result, such conversations lead to shared decision-making, improved patient-clinician communication, detection of overlooked problems, and tailored process monitoring thereby ensuring quality individualized care [4, 8–10].

The use of PCM data in healthcare has been a robust area of research at individual, organizational, and system levels around the world. Considerable emphasis has been placed on the use of aggregated PCM data to inform program evaluation, quality improvement, benchmarking, value-based healthcare, and to some degree managerial decision-making [11, 12]. Over several decades, developers and users have examined PROM applications to clinical practice (e.g., [6, 10, 13–16]). Although structures and processes exist to support the use of aggregated PCM data, the integration of individual-level PCM data by HCPs in daily clinical practice is challenging worldwide for various reasons, with calls for additional research to understand needs, influential factors, and best practices for effective implementation with a focus on end users [3, 17–20]. In the peer-reviewed literature about HPC provider use of PCMs in routine practice, eight systematic reviews [5, 10, 11, 21–25] and one scoping review [26] have published (see Additional file 1). While this number of systematic reviews would typically offer a robust body of evidence about the possible experiences of HCPs, upon a closer inspection most reviews predominantly reported on barriers and facilitators of PCM use [5, 10, 11, 22, 23, 25, 26] and five reviews limited PCM use to a specific practice setting (i.e., palliative care [5]; cancer care [10, 22, 26], and adult mental health [24]). Notably, Bantug and colleagues [11] differed from other reviewers in HCPs examination of effective methods for the interpretation of patient-reported data using various graphic displays. Although the reviews indicated they were about clinicians, only three studies specified selection criteria about the inclusion of HCPs [11, 21, 25]. These reviews mostly included a mixture of qualitative and/or quantitative research-designed studies. Only Gelkopf et al. [24] stipulated the inclusion of initiatives or projects (e.g., quality improvement and knowledge translation) exploring “real-world” PROMs implementation. In recent years, there have been increasing numbers of published quality improvement, organization implementation, and knowledge translation projects exploring PCM implementation in clinical practice. As such, there is a need to include peer-reviewed evidence about implementation projects that capture everyday experiences of end-users to advance our understanding of the practice behavioral changes (and struggles) faced by HCPs. To date, no single review has captured the voice of end-users, the HCPs, vis-à-vis their experiences of implementing individual-level PCM data in various practice settings to inform clinical decision-making and care planning. The apparent gap in knowledge justifies the need for our proposed systematic review.

This protocol builds on the work of the aforementioned reviews and will be informed by knowledge translation and implementation science theory. To this end, our aim is to conduct a comprehensive mixed methods systematic review (MMSR) to synthesize a wide range of evidence about the daily use of individual-level PCM data by HCPs in all practice settings or health services. The *P*opulation, Phenomenon of *I*nterest, *C*ontext (PIC) format (see Table 1) review question guiding the protocol is “How do healthcare providers implement individual-level patient-centered measurement/assessment tools (and the resultant data) as a routine part of their everyday practice?” This includes the following secondary review questions:

1. What are HCPs’ experiences in applying these tools in clinical practice?
2. How do HCPs interpret and integrate these tools to inform patient care?
3. What are the factors (barriers and facilitators) that influence PCM implementation by HCPs at the point of care?

**Table 1:** Definitions of the review question elements using the PIC format

PIC element	Definition
In this review, patients complete a PROM or PREM in a given practice setting/health service and then HCPs act on the resultant data in the provision of patient care.	
*P*opulation (P)	*Healthcare provider*(*s*) Refers to individuals from any health discipline or profession that provides direct health services to patients, clients, and/or families. HCPs are regulated or licensed healthcare professional; however, this may vary by country. HCPs may be referred to as clinicians. Common HCPs include but are not limited to registered nurses, nurse practitioners, physiotherapists, occupational therapists, physicians, social workers, dieticians, psychologists, pharmacists, and midwifes.
Phenomena of *i*nterest (I)	*Implement individual-level*, *patient-centered measurement/assessment tools* (*and the resultant data*) In a broad sense, *implementation* is the process or act of making something active or effective. In the context of PROMs, other synonyms include: employ, apply or application, utilize, use, integrate, interpret, draw on, make use of, and act on. The act of implementing PCMs in this review also captures the experiences, views, attitudes habits, practices, and routines of HCPs. *Patient-centered measurement* as an umbrella term refers to standardized assessment tools or questionnaires classified as PROMs or PREMs that every patient is eligible to complete. Thus, the questions are completed by the patient and are about outcomes that matter to them. The results are individual level numerical or textual data that indicate the patient’s current state or experience.
*C*ontext (C)	*Routine part of their everyday practice* Is an expression to describe an action that has been taken as a regular or common aspect of the HCP’s role. It is usually in relation to a HCP’s direct interaction between them and the patient/client/family. For example, in acute care or hospital settings, this term used maybe point-of-care or bedside where patient and providers interact on a regular basis, such as daily, weekly, or monthly. The context includes any practice setting or type of health service (e.g., acute care versus community care; private versus publicly funded, and community hospitals versus academic centers/teaching hospitals).

The protocol for this systematic review is part of a larger integrated knowledge translation (IKT), three-phase study to create user guidelines that support HPC use of PCM for clinical decision-making and care planning, wherein HCP interviews and a deliberative dialog, consensus-building forum with key stakeholders will also contribute to guideline development. The PCM implementation guidelines for HCPs will support routine collection, interpretation, and integration of these data in daily practice, ultimately contributing to effective, quality healthcare.

## Methods

The present protocol has been registered within the PROSPERO database (registration number CRD42020182904, November 3, 2020) and is being reported in accordance with the reporting guidance provided in the Preferred Reporting Items for Systematic Reviews and Meta-Analyses Protocols (PRISMA-P) statement [27] (see checklist Additional file 2). The review will be conducted in accordance with the Joanna Briggs Institute (JBI) methodology for mixed methods systematic review (MMSR) [28]. To find relevant research evidence, the *p*opulation, phenomenon of *i*nterest, and *c*ontext (PIC) format has been applied to formulate the review questions, devise a search strategy, and guide study selection criteria. The project will utilize an iterative process undertaken primarily by AW with input from members of the research staff and larger research team based on areas of expertise. A written record will be organized according to the matrix method [29].

### Information sources and search strategy

The proposed search will be conducted in accordance with the checklist for Peer Review of Electronic Search Strategies (PRESS) Guideline for systematic reviews [30] to achieve a balance of recall and precision. With academic librarian support (DD), the search strategy, including all identified keywords will be adapted for each database. An initial limited search of two databases (MEDLINE and CINAHL) was undertaken to identify articles on the topic. Since controlled vocabulary/subject headings are unique to each database, keywords have been identified as the most reliable approach for suitable recall. We determined that keyword searching, with the generation of all synonyms, plurals, and alternate spellings (e.g., centred and centered), for the phenomenon of interest (i.e., PCM and implementation) (see Additional file 3) produced high yield. Titles, abstracts, and keywords of relevant articles were used to assist in the identification of synonyms for each keyword. Although it is common to include a third keyword to represent the population or context elements of the PIC question, we found that it limited the precision of our results. As such, we decided to exclude a third concept for searching and instead include them as part of our selection criteria. Findings from our preliminary search informed the search for the project.

The evidence to answer our question will be retrieved by searching for the published literature between January 2009 onwards using eight databases with the EBSCO platform that covers the subjects of nursing, allied health, health sciences, psychological literature, physical therapy, occupational health, nutrition, kinesiology, and evidence-based reviews (i.e., MEDLINE, CINAHL, PsycINFO, Web of Science, Embase, SPORTDiscus, Evidence-based Medicine Reviews, and ProQuest (Dissertation and Theses)). We established a 10-year date range after a preliminary search of the literature noted that research about PCMs use at the individual level was first published around 2009. Upon further investigation, we determined that 71% of the relevant records were published from 2016 onwards. This confirmed our decision to use this search date. Limiters being used include: (a) scholarly/peer reviewed citations, (b) English language, and (c) date range. In the keyword search, we will used appropriate truncation (e.g., asterisks) and in some circumstances the boolean “NEAR” or proximity locators to link terms that may not be adjacent (e.g., barrier* n4 implement* and facilitat* n4 implement*) [31]. All identified search terms will be linked using Boolean operators. The boolean “OR” operator will be used to link search terms as a union for each concept for the purpose of expanding and broadening a search. The interaction of these concept searches will then be combined with “AND” to narrow the search [31]. A draft search strategy for CINAHL is provided in Additional file 4 as an example of search histories retained from all databases.

Our exploration will be supplemented by using other searching strategies to carry out a comprehensive search and counterbalance the limits of keyword database searching [31]. This includes footnote chasing (i.e., scanning the references of eligible articles), author searching of those publishing extensively in their field, and backward/forward citation searching of related systematic reviews and other seminal articles (e.g., large studies with numerous publications or those frequently cited) [31]. Additionally, a search of authors most frequently publishing in the field will be conducted. The ProQuest database will be used to search for eligible dissertations and theses. Upon completion of our database search, we will search the unpublished literature to lessen publication bias and to retrieve difficult to find literature and information regarding implementation projects. To avoid bias, we will use two approaches. First, a judicious examination of the “grey” literature (e.g., research reports, practice guidelines, and user guides) will be conducted using the Google© search engine from a university internet protocol address with the most cited search terms of the PIC concepts identified during our database searches. Second, we will access websites of credible organizations, agencies, and associations that may produce and publish knowledge translation documents supporting PCM implementation [31]. These websites will be identified by seeking the opinions of experts in the field.

### Types of evidence

The MMSR methodology combines an assortment of evidence to create a breadth and depth of understanding of the review questions posed to inform practice and policy. The inclusion of all available evidence, regardless of type, allows for the degree of agreement or discrepancies between sources of evidence as well as validating or triangulating the findings. Various aspects of a phenomenon of interest can be examined and the available data can contextualize the findings [28]. Furthermore, given that implementation at the point-of-care requires a variety of knowledge to inform practice, diverse evidence types will be sought. This review will consider peer-reviewed literature: quantitative, qualitative, and mixed methods studies in addition to reviews (i.e., systematic and literature), organizational implementation projects (e.g., quality improvement, knowledge translation project, implementation of PROMs, program evaluation, or pilot/feasibility project), and expert opinion (e.g., an individual, group or learned body that draws on their practical experience and understanding of the knowledge). We will include not only the evidence on the effectiveness of strategies for implementing PCM (“knowing *what*” type of evidence) but also evidence related to subjective experiences, attitude, behaviors, and/or the accepted discourse at the time of practice (“knowing *how*” type of evidence) [32]. Opinion-based evidence will be included when derived from expert opinions. That is, the opinions from experts in the field that were gained through some form of consensus building process (e.g., conference, think tank, special interest group, panel, and current discourse) [32]. Inclusion of the unpublished grey literature is unique to a systematic review of this nature.

### Selection criteria

The next step to finding relevant evidence for inclusion in this review is to define the selection criteria. To be included in the review, the literature needs to meet the following eligibility criteria (see Table 2): (a) HCPs in a clinical setting; (b) information pertaining to PROM or PREM implementation (e.g., HCP experiences, strategies for integrating into practice, influential factors, or attitudes toward use); (c) data were at the individual level; and (d) any study design. Articles will be excluded if (see Table 2): (a) articles focused exclusively on decision-makers (e.g., managers) or patients; (b) information pertaining to when and why PROMs and PREMs are used as well as impact of their use; (c) studies about instrument development, testing and selection; or (d) implementation of aggregated data. Studies with mixed samples (e.g., patients and HCPs) will be retained with the intent to extract and synthesize findings pertaining only to HCPs. Furthermore, to determine inclusion of studies in the review, we will apply the criteria in a specific order [34]. After each citation is confirmed as written in English and within the date limit, we will ensure it meets the study design criterion. Next, the phenomenon of interest criterion will be applied followed by screening for population and context.

**Table 2:** Selection criteria

Topic	Inclusion	Exclusion
*P*opulation (P)	• Healthcare providers	• Decision-makers exclusively • Patients exclusively
Phenomena of *i*nterest (I)	Studies about PREMs or PROMs and • experiences of applying or implementing • methods or strategies for integrating and interpreting (e.g., processes, logistics, tools, or workflow) • factors (barriers and facilitator) influencing implementation • views or attitudes toward their use	Studies about PREMs or PROMs and • impact or effectiveness • mechanisms by which they work (e.g., patient-provider communication) • ways used (e.g., screening, assessment, improve communication) • measurement development, testing, and selection • suitability for specific patient populations • a focus solely on patient-centered care
*C*ontext (C)	Studies concerning data at the individual (micro) level with patients: • routine clinical care • point-of care • everyday clinical practice • directly inform patient care or care planning • clinical decision-making • real-world application	Studies concerning aggregated data for purposes such as: • performance indicators or accreditation • value-based medicine • quality improvement or quality control • resource allocation, service provision, and economic evaluation • clinical registries • reimbursement and payer issues • benchmarking • drug development
Study design	Published scholarly work including research, pilot or feasibility projects, evidence-based implementation/quality improvement, systematic reviews, literature reviews, and expert opinion	Published literature such as editorials, opinion or position papers, commentary, study protocols, conference proceedings or abstracts, and theory. Insufficient information reported on study design

### Study selection procedure

Following the completion of these searches, all identified citations will be loaded into EndNote X9© (Version 9.3.3) [33] and duplicates removed. AW will provide a thorough orientation to those involved in the selection process to ensure rigor. Given the large quantity of records anticipated to be retrieved (e.g., greater than 20000), the first 100 record titles and abstracts will be screened by two independent reviewers for assessment against the inclusion criteria to be identified as relevant, not relevant, or maybe relevant. Following that, AD will screen all other records to determine relevancy. AW to confirm eligibility will rescreen those identified as potentially relevant. To ensure validity of the selection criteria, in the EndNote© library for this project, AW will conduct keyword searches (e.g., outcome measure, patient outcome, patient-reported) of record titles of those deemed irrelevant to reapply the selection criteria. Finally, all relevant studies will be retrieved in full text and their citation details independently reviewed (AW and AD) against the selection criteria to confirm inclusion. Reasons for further exclusion of all studies will be recorded. Any disagreements that arise between the reviewers at each stage of the study selection process will be resolved through discussion [29, 34]. A PRISMA diagram [35] showing details of studies included and excluded at each stage of the study selection process will be created.

### Assessment of methodological quality

Critical appraisal of included studies will determine the level of evidence and methodological quality as a basis for our confidence to act on the recommendations from our synthesis. Two independent reviewers, blinded to each other’s assessments, will retrieve all included citations, and applicable supplemental files, in full-text format for assessment. AW will provide a robust orientation to primary reviewers (AD, DG, SH, FH, LE, SL, LM, and two undergraduate research assistants) of the process and appraisal checklists to ensure rigor. Authors will be contacted to request missing or additional data for clarification, where required. Any disagreements that arise between the reviewers will be resolved through consensus discussions among select team members, or a blinded third reviewer.

We will use the following standardized JBI critical appraisal instruments for assessing quality (see Table 3): systematic review, qualitative, cross-sectional, prevalence, case report, and text and opinion [39, 41]. To evaluate organizational implementation projects, three questions from the JBI case report checklist [42] were combined with questions from the Johns Hopkins’ organizational experience checklist for non-research evidence [40] and questions for quality improvement interventions [43, 44]. Similarly, the JBI checklists for analytical cross-sectional [42] and prevalence survey [45, 46] studies were modified to include four additional questions about the research questions, research methods, ethical approval, and justified conclusions [47–49]. JBI checklists do not exist for mixed methods studies or literature reviews. AW conducted an extensive review of the literature to locate other standardized tools of high reliability and validity. Based on a parsimonious set of core criteria, the mixed method checklist focuses on both the effective integration of the quantitative and qualitative components of studies, as well as the provision of a rationale for using a mixed methods design [38, 50, 51]. The checklist used for literature reviews will be based on the Johns Hopkins’ form for non-research evidence [40].

**Table 3:** Summary of the critical appraisal checklists by research design

Type of evidence	Critical appraisal
Systematic review	JBI Systematic Review Appraisal Tool [36]
Qualitative	JBI Qualitative Appraisal Tool [37]
Analytical cross-sectional	JBI Analytical Cross Sectional Appraisal Tool with others
Survey	JBI Prevalence Appraisal Tool in combination with others
Mixed method	Mixed Method Appraisal Tool [38]
Organizational implementation	JBI Case Report with others
Expert opinion	JBI Text and Opinion Appraisal Tool [39]
Literature review	JH Non-research Evidence (Literature Review) Appraisal Tool [40]

All checklists contain a series of criteria (range 8 to 15 questions) scored as being “met” or “not met” or “unclear” and, in some instances, as “not applicable.” Following critical appraisal, all studies will be given a percentage score with higher scores indicating a greater percentage of the quality criteria were met. The research team decided not to set a quality threshold to exclude evidence. Rather, once the data are synthesized, we will determine the confidence to act based on the quality and level of the evidence. A modified version of the JBI levels of evidence [52, 53] for meaningfulness will be used as it best aligns with our review questions and the nature of the evidence. The five levels are as follows:

1. Quantitative or mixed-methods systematic review
2. Qualitative or mixed-methods synthesis and single experimental-based quantitative study
3. Single qualitative and descriptive or observational quantitative study
4. Systematic review of expert opinion and organizational implementation project single study (e.g., evidence-based practice, quality improvement, and knowledge translation)
5. Expert opinion and literature review

Given the evidence in this review is explorative, descriptive, and interpretative in nature, the JBI Grades of Recommendation will be the criteria used to define the overall strength of the recommendation (i.e., strong or weak) [53, 54].

### Data extraction

The data extraction step provides the means by which the most pertinent information about the topic (i.e., study characteristics and findings) can be summarized and culled from the primary studies. All source documents will be loaded into the data management software NVivo^TM^ (Version 12.6) [55]. Using this software, a review matrix will be generated to maximize efficiency and create “order out of chaos” ([29], p. 150). Column topics for the matrix will be defined according to the purpose of the proposed systematic review to capture pertinent bibliographic information, methodological characteristics, and content-specific characteristics (e.g., implementation theory) of each included citation (see Table 4) [29]. Column topics for which there is a discrete response option (e.g., methodology) will be extracted using the NVivo^TM^ file classification function. The NVivo^TM^ codes function will be used to identify the column topic response for items that have more than one response option (e.g., studies conducted in multiple settings or involving multiple HCPs). These data will offer contextual and methodological data to support the data synthesis results [29]. Select team members will be involved in assembling the extracted data from all included articles with relevant accompanying illustrations (e.g., participant quotes or statistical test values). Notes on the definition of column topics and response options as well as the overall extraction process will be kept to ensure consistency among extractors.

**Table 4:** Bibliographic information and study attributes to be abstracted

Bibliographic information	Study attributes
• Authors • Year of publication • Article title • Keywords • Digital object identifiers	• Country/ies of study • Methodology • Research design • Implementation theory • Health service • Practice setting • Sample population/profession of healthcare providers • Sample size • Sampling method • Level of evidence • PROM and PREM instruments used

The next step will be the extraction of the pertinent study findings, specifically from the results and discussion sections of each citation. Using NVivo^TM^ [55], the process of synthesis begins as the study findings will be extracted into specific codes. All study findings from the included citations will be coded for analysis as textual descriptions. Qualitative data will be composed of themes or subthemes with corresponding illustrations (e.g., quotations, tables, and figures). The quantitative data (e.g., descriptive or inferential statistics) will be converted into “qualitized data.” This process will involve the transformation of all quantitative data into textual descriptions or narrative interpretation in a way that answers the review questions. When necessary, corresponding statistical test results can be captured as part of the coding process. As per the narrative synthesis approach [56], code names will be based on a theoretical framework. In our study, we will use the Consolidated Framework for Implementation Research (CFIR) [57]. The CFIR is an evidence-based framework used to assess multiple contexts and identify factors that might influence the process and effectiveness of the implementation of a specific intervention, which, in our review, is PCM. The five major domains are intervention characteristics (8 items), inner setting (5 items), outer setting (4 items), characteristics of individuals involved (5 items), and implementation process (4 items) [57]. A further framework will be used to code the identified implementation processes or actions to support a practice change. For this, we will use the validated Expert Recommendations for Implementing Change (ERIC), which is a compilation of 73 discrete strategies in nine clusters [58, 59]. Codes not represented in either framework will be created, as determined, by AW to answer the review questions. In this manner, extraction and initial synthesis occur simultaneously. To reduce coding error during data extraction, we will develop a coding protocol, provide coder training, leverage our substantive expertise among team members, and use the NVivo coding comparison feature to improve reliability [31]. In summary, the overall extraction process of transforming and coding these data will facilitate each element of the narrative synthesis to integrate the existing evidence and answer the review questions [28].

### Data synthesis

The synthesis will follow a convergent integrated approach as per the JBI methodology for MMSR. In this manner, data from all types of evidence will be simultaneously extracted and synthesized into meaningful codes. Furthermore, this integrated approach means that the transformed “qualitized” data will be combined to identify patterns across all the studies as well as explore relationships of the data between and within the studies [28]. The integration of these data will be guided by a narrative synthesis approach [56], which is well suited for MMSR that utilize diverse types of evidence and has sample heterogeneity [28]. Moreover, this approach allows for the use of theoretical frameworks to shape the analysis. In our case, the analysis will use two implementation science frameworks allowing us to focus broadly on the implementation process as well as effective strategies to implement and sustain changes in HCP’s behavior. Popay et al. [56] identifies four iterative elements to a narrative synthesis.

- Element 1: *The role of theory in evidence synthesis.* Contributing to knowledge translation theory on how PCM implementation works, why, and for whom we will use the CFIR [57] and ERIC strategies [58, 59] that are based on theories of change. With the use of NVivo^TM^ for extraction, the process of synthesis begins as the theory contributes to the interpretation of study findings and determines how widely applicable the findings may be. Study data will be grouped into discrete constructs according to the CFIR domain about the characteristics of the end-users; NVivo^TM^ refers to these as codes. In this way, theory building and theory testing can be incorporated as a key aspect of the proposed systematic review [56].
- Element 2: *Developing a preliminary synthesis*. A preliminary synthesis is conducted to understand the codes identified and summarize the results of included studies. This will be achieved by defining patterns of findings simultaneously across all the studies based on our primary and secondary questions. An initial description of the findings will evolve based on similarity in meaning to produce an integrated synthesis. One tool used is grouping and clustering [56]. Using NVivo^TM^ to code the data within each citation will subsequently allow us to visualize prominent theoretical constructs. As per the narrative synthesis approach, we next identify the main, recurrent, and/or most important themes across the aggregated data from multiple studies. This will be done in a staged, iterative approach starting with the highest to lowest level of evidence. For example, we will code all systematic review studies to create NVivo^TM^ coding summary reports for each construct. Reading the aggregated data report allows us to identify descriptions of salient themes between and within each theoretical construct from this group of studies. Next, we will code the quantitative studies, read the coding summary report to identify salient themes and add the cumulative description. After that, we move to the next level of evidence to repeat the process. An outcome of this element is a summary of the salient themes across studies.
- Element 3: *Exploring relationships of the data between and within the studies.* The purpose of the third element is to identify reasons that might explain any differences in the findings regarding the experiences of HCPs. The emerging patterns identified in the pooled data will be further analyzed to identify factors, study characteristics, and context explaining differences. Comparing and contrasting relationships across studies is important to this stage of the synthesis as a means to explore the influence of heterogeneity. Possible tools for consideration are subgroup analysis and mind mapping [56]. This will allow us to examine patterns in the data related to the general as well as the particular (e.g., HCP group, sector, and practice setting) associated with PCM use. The NVivo^TM^ relationship and query features will aid in our exploration of associations.
- Element 4: *Assessing the robustness of the synthesis*. This element allows for the integration of the quality assessments to determine the strength of the evidence and support with the trustworthiness of the synthesis products (e.g., answers to the study questions and recommendations). Using NVivo^TM^, the included studies will be assigned both a level of evidence and a quality score that will be cross-linked to the products of the synthesis. From this, a final determination of the strength of the evidence to support conclusions draw from the synthesis process can be made [56].

#### Integration of the evidence and dissemination

Using an IKT approach, our 30-member team includes researchers, knowledge users (KU), and patient partners from four practice settings to represent various degrees of PCM implementation and use by diverse HPCs. The described MMSR products (phase 1) will be integrated with findings from HCP interviews (phase 2) conducted to illicit “real world” experiences in the four KU practice settings. These data will comprise a research brief for use in the final phase consisting of a deliberative dialog, consensus-building forum with key stakeholders to triangulate and reach consensus about the topic of study. Taken together, these three phases support the robust development of an evidence-based guideline on how to interpret and “act on” PCM data to inform clinical decision-making and care planning that is patient-centered. The guideline will be applicable to individual-level PMC data use in all practice settings (general) for adaptation to the particular (local context). This approach is used to ensure that the resultant SR findings, and subsequent guideline, are relevant and applicable to audiences at various levels: healthcare providers (micro); healthcare managers/leaders (meso) and decision-makers (meso or macro) responsible for PCM implementation; researchers (meso); and educators of entry-level health professional programs (meso).

Reporting for this study will follow the PRISMA statement [60]. Any amendments from the original review protocol when conducting the review will be outlined in PROSPERO and reported in the final manuscript. Traditional dissemination methods will be used to report the MMSR findings in relevant, peer-reviewed journals and reputable conferences. The findings of this review form the basis of the final guideline document (print-based and open-source version) to be made available to global audiences through various knowledge translation activities. First, a virtual dissemination event at the end-of-grant will be held to launch the guideline. Accompanying the guideline will be a one-page summary of key take-home messages to increase influence and relevance of the guideline to local contexts. In alignment with the IKT approach, members of the research team will support the disseminating via suitable local channels to facilitate guideline uptake. Finally, the guideline will be made readily available to applicable grey literature sites, promoted via social networks, and posted on a suitable website for ongoing access via the World-Wide Web. These guidelines can be used in conjunction with other user guides for the implementation of PCM in clinical practice [7, 61–64].

## Discussion

The aim of the proposed MMSR using narrative synthesis is to address an existing gap about the needs, capabilities, motivations, and individual factors influencing HCPs adoption behavior (i.e., integration of individual-level PCM data into daily practice for decision-making and care planning). The evidence on implementing PCM focuses predominantly on the CFIR domains about the intervention characteristics (e.g., [7, 61, 64]), inner setting, and implementation process [63]. To date, no single review captures the voice of end-users. This knowledge is essential to subsequently determine effective methods/strategies for both initial and sustained PCM implementation by HCPs.

Building on existing evidence, strengths of the proposed project are the inclusion of all practice settings and all HCP disciplines. Furthermore, the protocol methods were selected to mitigate the limitations of past reviews and broaden our understanding of this phenomenon [30, 31, 41]. For example, we created a comprehensive list of search terms/keywords to capture the diverse terminology for PCM and implementation that is used in a range of health services and practice settings. The use of these keywords in conjunction with numerous databases relevant to the topic of interest will result in a higher yield of relevant citations from which to draw conclusions [31]. Careful consideration about the types of primary literature to include was taken as to adequately represent the scope and complexity of HCPs’ implementation experiences [28, 32]. A final strength of our project is the application of a theory-based framework to the cumulative body of evidence. Results can inform the adoption of theory-based behavioral change strategies that align with the characteristics and experiences of end-users.

While there are several strengths to this systemtatic review, we also anticipate various challenges and limitations. One particular challenge will be determining whether the CFIR domain “characterises of individuals” has sufficient constructs to capture the scope of HCP experiences, needs, and influential factors found in the literature. Based on the anticipated large body of eligible literature, the narrative synthesis will be complex and arduous, particularly element 3. A further challenge will be managing the larger yield in a timely manner to ensure it is current while triangulating it with the interviews and deliberative dialog before developing the final guideline. The study is confronted with selection bias resulting from the following: (a) the restriction of the literature search from January 2009 onwards; (b) the inclusion of evidence published only in the English language; and (c) the exclusion of search terms for the names of specific patient-reported instruments or tools (e.g., quality of life). To mitigate selection bias, we created explicit selection criteria that were based on the review questions to determine eligible literature [34]. Most of the evidence in this is explorative, descriptive, and interpretative in nature. Although not considered high on the hierarchy of evidence pyramid, it is the best evidence currently available. In the final element of the narrative synthesis approach, a final determination of the strength of the recommendations will be made.

In summary, the protocol for this MMSR meets an internationally clinically driven need to conduct a comprehensive synthesis of various types of evidence on (a) experiences of HCPs applying these tools, (b) effective methods for HCP interpretation and integration of individual-level PCM data, and (c) identification of relevant factors influencing PCM implementation. This rigorous systematic review is one part of a larger three-phased project that will be followed by HCP interviews and a consensus-building forum to elicit input from the end-users who are asked to change their practice behaviors. The wealth of evidence obtained from this review will inform the implementation of PCM as a complex intervention requiring synergy among the five CFIR domains to support optimal implementation and sustainability in the use of PCM data. The outcome of this review will provide knowledge users with practical, actionable, and evidence-based information. Overall, this project contributes to a larger study with the goal to develop of an evidence-informed guideline that supports the use of effective implementation methods/strategies to enhance the widespread incorporation of PCM data into HCPs’ daily practice. Overall, this project is intended to promote “shared ownership” of individual-level PCM data to better understand and address what matters to patients.

## Supplementary Information

**Additional file 1.** Summary of Published Systematic and Scoping Reviews about Healthcare Providers Using PCM in Routine Practice.

**Additional file 2.** PRISMA-P Checklist.

**Additional file 3.** Master List of Synonyms for Key Concepts in the Search Strategy.

**Additional file 4.** Example of Search Strategy in Bibliographic Database.

## Data Availability

Not applicable.

## Abbreviations

CBOs: Clinician-based outcomes
CFIR: Consolidated Framework for Implementation Research
CINAHL: Cumulative Index to Nursing and Allied Health Literature
EBSCO: Elton B. Stephens Company
EMBASE: Excerpta Medica Database
ERIC: Expert Recommendations for Implementing Change
HCP: Healthcare provider
IKT: Integrated Knowledge Translation
JBI: Joanna Briggs Institute
KU: Knowledge Users
MEDLINE: Medical Literature Analysis and Retrieval System Online
PCM: Patient-centered measurement
PIC: Population, phenomenon of interest, and context
PICO: Population, issue/interest, context/comparison, and outcome
PREMs: Patient reported experience measures
PRESS: Peer Review of Electronic Search Strategies
PRISMA: Preferred Reporting Items for Systematic Review and Meta-Analyses
PRISMA-P: Preferred Reporting Items for Systematic Review and Meta-Analyses Protocols
PROs: Patient-reported outcomes
PROMs: Patient-reported outcome measures
PsycINFO: Psychological index
ROM: Routine outcome monitoring
SPORTDiscus: None available
