# Supplementary material for: Healthcare provider characteristics that influence the implementation of individual-level patient-centered outcome measure (PROM) and patient-reported experience measure (PREM) data across practice settings: a protocol for a mixed methods systematic review with a narrative synthesis
Source: Syst Rev. 2021 Jun 9;10:169. doi: 10.1186/s13643-021-01725-2 (PMC8188663; doi:10.1186/s13643-021-01725-2)
Supplement: Supplementary file 1 — Additional file 1. Summary of Published Systematic and Scoping Reviews about Healthcare Providers Using PCM in Routine Practice. [file 13643_2021_1725_MOESM1_ESM.docx]

Additional File 1: Summary of Published Systematic and Scoping Reviews about Clinicians Using PCM in Routine Practice (file extension .docx)

Below is a summary of the nine most relevant reviews about PCM data use by HCP at the individual-level.

| Author Name in Chronological Order | Objectives | Phenomenon of Interest Pertinent to this Review  And  Clinical Area | Types of Evidence Included  and  Synthesis Method | Databases Searched and  Search Dates | Search Terms for Patient-Reported Measures | Included # Articles (Search Results) | Inclusion Criteria Pertaining to HCPs | Quality or Bias Assess-ment Conducted |
| --- | --- | --- | --- | --- | --- | --- | --- | --- |
| Duncan 2012^2^ (25) | What are the barriers and facilitators to routine outcome measurement by allied health professionals in practice? (p. 2) | Barriers and facilitators to routine outcome measurement  Area: Any setting | No restriction on study design  Narrative synthesis | MEDLINE, CINAHL, PsycINFO  ≤ 2010 | 13 synonyms | 15 (960) | Concerned  with identifying or researching factors which  acted as facilitators and/or barriers in the routine use of  outcome measures by allied health professionals in practice. | Yes |
| Antunes, 2014^1^ (5) | 1. Identify barriers and facilitators to the systematic implementation of PROMs in palliative care clinical practice.  2. Identify needs and other comments of clinical teams regarding the routine use of  PROMs.  3. Identify lessons learned on the process of implementation of PROMs in clinical practice (p. 160). | Barriers and facilitators to implementation of PROMs  Lessons learning on the PROMs implementation process  Area: Palliative | All study designs  Narrative synthesis | MEDLINE, CINAHL, PsycINFO, Embase, British Nursing Index  1985-Mar 2011 | Example for British Nursing Index  Outcome measure*  PROs  PROMs  Patient reported outcome?  Patient reported outcome measure?  Self-reported measure?  (Appendix 1) | 31 (3863) | Not stated | Yes |
| Boyce 2014^1^ (11) | Investigated the experiences of healthcare professionals with the use of PROMs . . . . and to synthesise findings about the barriers and facilitators to their use (p. 509). | Use of PROMs  Barriers and facilitators of PROMs use  Area: Any setting | Qualitative  Thematic analysis | PubMed, CINAHL, PsycINFO  ≤ Aug 2013 | 12-15 synonyms depending on database | 16 (8344) | Healthcare professionals.  Professionals’  views of PROMs after receiving PROMs feedback  about individual patients or groups of patients. | Yes |
| Howell 2015^1^ (26) | 1. Which PROMs does the published, English literature show have been implemented for use in routine cancer clinical practice and in what phases of the trajectory?  2. What are the barriers and enablers influencing clinical uptake of PROMs in routine care?  3. What is impact of the routine use of PROMs on outcomes at the patient, provider, and system levels? (p. 1846-47) | Barriers and enablers influencing clinical update of PROMs  Area: Cancer/oncology | Quantitative, qualitative, or systematic literature reviews  Scoping review | MEDLINE, CINAHL, PsycINFO  2003-2013 | Examples given included  self-report questionnaire  self-assessment  PRO  PROMs  PROMIS  Outcomes | 30 (2447) | Evaluated outcomes at the patient, clinical practice, or care process or system-level or barriers/  enablers to the uptake or use of PROMs | Not specified |
| Bantug 2016^1^ (21) | Review existing literature examining the interpretation of PRO data displayed graphically (p. 484) | Interpretation of PRO data  displayed graphically  Area: Any setting | Quantitative or qualitative  Integrative approach | MEDLINE  1999-2014 | Patient-reported outcome  routine clinical care  health-related quality of life  health outcomes (p. 485) | 9 (242) | Group- or individual-level PRO data for clinicians or patients | Not specified |
| Foster 2018^1^ (23) | To conduct a systematic review of reviews to identify the facilitators and barriers to implementing PROMs in organisations delivering health related services (p. 2-3) | Facilitators and barriers to impacting the implementation of PROMs  Area: Any setting | Systematic reviews  Framework synthesis | MEDLINE, CINAHL, PsycINFO, Embase, Cochrane Database of Systematic Reviews  ≤ 2017 | PROM, PROMS, patient reported outcom*, PROs, PRO, PREM, PREMs, patient reported experience measure*, ePROs, ePRO, or outcome measure* | 6 (2047) | Focus on health-related services irrespective of the type of provider. | Yes |
| Yang 2018^1^ (10) | 1. Who  uses PROs as a tool to improve communication with patients and which topics are discussed?  2. Through what mechanisms do PROs facilitate communication between clinicians and patients?  3. What factors act as barriers or facilitators to PRO use and improved patient-clinician communication? (p. 42) | Barriers or facilitators to PRO use  Area: Cancer/oncology | Not specified  Realist synthesis | MEDLINE, CINAHL, PsycINFO, Embase, Cochrane Database of Systematic Reviews, Cab Direct  ≤ 2016 | Example MEDLINE  PROM  PROMs  PROMIS  patient reported outcome*  patient* outcome* measure*  self report adj1 (questionnaire or measure)  Exp *Health Status Indicators/  Questionnaires/  *Psychometrics/  Self Report/  Health Surveys/ | 43 (610) | PROs were used as an intervention, not as a study outcome  measurement tool | Yes |
| van Egdom 2019^1^ (22) | 1. How have PROMs been administered in breast cancer care?  2. What is the impact of PROM administration on patients, care providers, and healthcare services or processes?  3. What are the facilitators and barriers that influence the integration of PROM  collection in routine breast cancer clinical practice? (p. 1198) | Facilitators and barriers that influence the integration of PROM  Collection  Area: Cancer/oncology | Excluded case report, editorial, review or study protocols  Not specified | MEDLINE, CINAHL, PsycINFO, Embase, Cochrane Central, Web of Science  ≤ 2017 | patient-reported outcome'/de OR 'patient reported outcome measure'/de OR (('self report'/de) AND ('outcome assessment'/de OR 'quality of life'/exp OR 'quality of life assessment'/exp OR 'complication'/de OR 'symptom'/de OR 'wellbeing'/de OR 'psychological well-being'/de OR 'health status'/de )) OR PROM OR PROMs):ab,ti) | 34 (2311) | No HCP criteria specified | Yes |
| Gelkopf 2020^1,2^ (24) | 1. To ascertain the goals of the PROM/ROM projects.  2. To identify the setting, diagnoses and services provided in PROM/ROM  projects.  3. To identify the instruments used to measure outcome,  the domains measured and the process that led to the choice of instruments.  4. To gather information on the frequency of evaluation and the methods of data collection.  5. To gain information about the feasibility of ROM through the follow-up data collection rate; the training needs and type of feedback provided.  6. To gather  information on the barriers and limitations encountered to implement ROM and ways to overcome those barriers (p. 2). | Barriers and limitations encountered to implement  ROM  Area: Adult mental health | Peer-reviewed publications including specific initiatives or implementation of PROM data  Unnamed, categories with consensus and triangulation | PsycINFO  PubMed (including MEDLINE, Biomed Central, Embase Psychiatry, Elsevier’s Science Direct) (cited by Roe, Mazor & Gelkopf, 2019)  2000-2018 | 14 synonyms (cited by Roe, Mazor & Gelkopf, 2019) | 103 (19589) | Exclusion of articles on the views, knowledge, attitudes and perceptions of consumers, providers, or other stakeholders regarding the use of  PROMs/ROMs. | Not specified |

^1^PROMs or PROs

^2^Routine outcome monitoring
