# Supplementary material for: Healthcare provider characteristics that influence the implementation of individual-level patient-centered outcome measure (PROM) and patient-reported experience measure (PREM) data across practice settings: a protocol for a mixed methods systematic review with a narrative synthesis
Source: Syst Rev. 2021 Jun 9;10:169. doi: 10.1186/s13643-021-01725-2 (PMC8188663; doi:10.1186/s13643-021-01725-2)
Supplement: Supplementary file 3 — Additional file 3. Master List of Synonyms for Key Concepts in the Search Strategy. [file 13643_2021_1725_MOESM3_ESM.docx]

**Additional File 3: Master List of Synonyms for Key Concepts in the Search Strategy** (file extension .docx)

Keyword searching will include search terms from the two key concepts in the PIC-derived review question: patient-centred measurement and implementation. This list contains all generated synonyms, plurals, and alternate spellings.

**Patient-Centred Measurement**

"family caregiver* assess*" OR

"family caregiver* index*" OR

"family caregiver* indicator*” OR

"family caregiver* instrument*" OR

"family caregiver* inventor*" OR

"family caregiver* measur*" OR

"family caregiver* outcome*" OR

"family caregiver* questionnaire*" OR

"family caregiver* scale*" OR

"family caregiver* screen*" OR

"family caregiver* survey*" OR

"family experience assess*" OR

"family experience index*" OR

"family experience indicator*” OR

"family experience instrument*" OR

"family experience inventor*" OR

"family experience measur*" OR

"family experience outcome*" OR

"family experience questionnaire*" OR

"family experience scale*" OR

"family experience screen*" OR

"family experience survey*" OR

“family outcome assess*" OR

"family outcome index*" OR

"family outcome indicator*” OR

"family outcome instrument*" OR

"family outcome inventor*" OR

"family outcome measur*" OR

"family outcome questionnaire*" OR

"family outcome scale*" OR

"family outcome screen*" OR

“family outcome survey*” OR

"family-reported experience questionnaire*" OR

"family-reported outcome measur*" OR

"family satisfaction assess*" OR

"family satisfaction index*" OR

"family satisfaction indicator*” OR

"family satisfaction instrument*" OR

"family satisfaction inventor*" OR

"family satisfaction measur*" OR

"family satisfaction outcome*" OR

"family satisfaction questionnaire*" OR

"family satisfaction scale*" OR

“family satisfaction survey*” OR

"Health care outcome* assess*" OR

"Health care outcome* index*" OR

"Health care outcome* indicator*” OR

"Health care outcome *instrument*" OR

"Health care outcome* inventor*" OR

"Health care outcome* measur*" OR

"Health care outcome* questionnaire*" OR

"Health care outcome* scale*" OR

"Health care outcome* screen*" OR

"Healthcare outcome* survey*" OR

"Healthcare outcome* assess*" OR

"Healthcare outcome* index*" OR

"Healthcare outcome* indicator*” OR

"Healthcare outcome* instrument*" OR

"Healthcare outcome* inventor*" OR

"Healthcare outcome* measur*" OR

"Healthcare outcome* questionnaire*" OR

"Healthcare outcome* scale*" OR

"Healthcare outcome* screen*" OR

"Healthcare outcome* survey*" OR

"Health outcome assess*" OR

"Health outcome index*" OR

"Health outcome indicator*” OR

"Health outcome instrument*" OR

"Health outcome inventor*" OR

"Health outcome measur*" OR

"Health outcome questionnaire*" OR

"Health outcome scale*" OR

"Health outcome screen*" OR

"Health outcome survey*" OR

"health status assess*" OR

"Health status index*" OR

"Health status indicator*” OR

"health status instrument*" OR

"Health status inventor*" OR

"Health status measur*" OR

"Health status outcome*" OR

"Health status questionnaire*" OR

"Health status scale*" OR

"Health status screen*" OR

"Health status survey*" OR

“Patient-centred experience” OR

"Patient-centered experience" OR

“Patient-centred outcome*" OR

"Patient-centered outcome*" OR

“Patient experience assess*" OR

"Patient experience index*" OR

“Patient experience indicator*” OR

"Patient experience instrument*" OR

"Patient experience measur*" OR

"Patient experience outcome*" OR

"Patient experience questionnaire*" OR

"Patient experience screen*" OR

"Patient experience scale*" OR

"Patient experience survey*" OR

“Patient outcome* assess*" OR

"Patient outcome* index*" OR

“Patient outcome* indicator*” OR

"Patient outcome* instrument*" OR

"Patient outcome* inventor*" OR

"Patient outcome* measur*" OR

"Patient outcome* questionnaire*" OR

"Patient outcome* screen*" OR

"Patient outcome* scale*" OR

"Patient outcome* survey*" OR

“Patient-orientated assess*" OR

"Patient-orientated index*" OR

“Patient-orientated indicator*” OR

"Patient-orientated instrument*" OR

"Patient-orientated measur*" OR

"Patient-orientated questionnaire*" OR

"Patient-orientated scale*" OR

"Patient-orientated survey*" OR

“Patient-oriented assess*"

"Patient-oriented index*"

“Patient-oriented indicator*”

"Patient-oriented instrument*"

"Patient-oriented inventor*"

"Patient-oriented measure*"

"Patient-oriented questionnaire*"

"Patient-oriented scale*"

"Patient-oriented screen*"

"Patient-oriented survey*"

"patient-reported experience*" OR

"patient-reported outcome*" OR

“patient-reported satisfaction*” OR

"patient satisfaction assess*" OR

"patient satisfaction index*" OR

"patient satisfaction indicator*” OR

"patient satisfaction instrument*" OR

"patient satisfaction inventor*" OR

"patient satisfaction measur*" OR

"patient satisfaction outcome*" OR

"patient satisfaction questionnaire*" OR

"patient satisfaction scale*" OR

"patient satisfaction screen*" OR

“patient satisfaction survey*” OR

“People-centred assess*" OR

"People-centred index*" OR

"People-centred instrument*" OR

"People-centred measur*" OR

"People-centred outcome*" OR

"People-centred screen*" OR

"People-centred scale*" OR

"People-centred survey*" OR

“patient-centred healthcare outcomes” OR

“patient-centred health care outcomes” OR

“patient-centered healthcare outcomes” OR

“patient-centered health care outcomes” OR

“People-centered assess*" OR

"People-centered index*" OR

“People-centered indicator*” OR

"People-centered instrument*" OR

"People-centered measur*" OR

"People-centered outcome*" OR

"People-centered screen*" OR

"Person-centered outcome*" OR

"Person-centred outcome*" OR

"person-reported outcome*" OR

"person-reported experience*" OR

"PREM" OR

“PREMs” OR

"PROM" OR

"PROMs" OR

"PROMIS"

**Implementation**

"Attitudes of health personnel" OR

Barrier* n4 implement* OR

challeng* n4 implement* OR

"Clinical application*" OR

"clinical decision making" OR

"Clinical decision support*" OR

"clinical educat*” OR

"clinical support*” OR

"clinician attitude*” OR

"clinician perception*” OR

"clinician view*” OR

"computer application*" OR

"electronic application*" OR

Enabler* OR

Experience* OR

facilitat* n4 implement* OR

“health personnel attitude” OR

"Implementation science" OR

Implement* OR

integrat* OR

Interpret* OR

"Knowledge translat*" OR

"online application*" OR

"Practice guid*" OR

"Practise guid*" OR

"Practic* application*" OR

"practice educat*” OR

"practice support" OR

"Professional practice" OR

"Professional development" OR

"program evaluat*” OR

"provider attitude*” OR

"provider perception*” OR

"provider view*” OR

"quality improve*" OR

"smartphone application*" OR

"stakeholder engagement” OR

"Training project” OR

"Training program” OR

"training clinician*” OR

"web* application*"
