# Supplementary material for: Healthcare provider characteristics that influence the implementation of individual-level patient-centered outcome measure (PROM) and patient-reported experience measure (PREM) data across practice settings: a protocol for a mixed methods systematic review with a narrative synthesis
Source: Syst Rev. 2021 Jun 9;10:169. doi: 10.1186/s13643-021-01725-2 (PMC8188663; doi:10.1186/s13643-021-01725-2)
Supplement: Supplementary file 4 — Additional file 4. Example of Search Strategy in Bibliographic Database. [file 13643_2021_1725_MOESM4_ESM.docx]

**Additional File 4: Example of Search Strategy in Bibliographic Database** (file extension .docx)

Draft of search strategy to be for CINAHL database with year and language limits.

Database name: CINAHL Complete

Date searched: 2019-12-10

Limiters: Human

Language: English

Type of document: peer reviewed, academic journals

Date range: January 1, 2010 to December 31, 2020

| **Implementation** | **AND** | **PCM** |
| --- | --- | --- |
| "Attitudes of health personnel" OR Barrier* n4 implement* OR challeng* n4 implement* OR "Clinical application*" OR "clinical decision making" OR "Clinical decision support*" OR "clinical educat*" OR "clinical support*" OR "clinician attitude*" OR "clinician perception*" OR "clinician view*" OR "computer application*" OR "electronic application*" OR Enabler* OR Experience* OR facilitat* n4 implement* OR "health personnel attitude" OR "Implementation science" OR Implement* OR integrat* OR Interpret* OR "Knowledge translat*" OR "online application*" OR "Practice guid*" OR "Practic* application*" OR "practice educat*" OR "practice support" OR "Practise guid*" OR "Professional practice" OR "Professional development" OR "program evaluat*" OR "provider attitude*" OR "provider perception*" OR "provider view*" OR "quality improve*" OR "smartphone application*" OR "stakeholder engagement" OR "Training project" OR "Training program" OR "training clinician*" OR "web* application*" |  | "family caregiver* assess*" OR "family caregiver* index*" OR "family caregiver* indicator*" OR "family caregiver* instrument*" OR "family caregiver* inventor*" OR "family caregiver* measur*" OR "family caregiver* outcome*" OR "family caregiver* questionnaire*" OR "family caregiver* scale*" OR "family caregiver* screen*" OR "family caregiver* survey*" OR "family experience assess*" OR "family experience index*" OR "family experience indicator*" OR "family experience instrument*" OR "family experience inventor*" OR "family experience measur*" OR "family experience outcome*" OR "family experience questionnaire*" OR "family experience scale*" OR "family experience screen*" OR "family experience survey*" OR "family outcome assess*" OR "family outcome index*" OR "family outcome indicator*" OR "family outcome instrument*" OR "family outcome inventor*" OR "family outcome measur*" OR "family outcome questionnaire*" OR "family outcome scale*" OR "family outcome screen*" OR "family outcome survey*" OR "family-reported experience questionnaire*" OR "family-reported outcome measur*" OR "family satisfaction assess*" OR "family satisfaction index*" OR "family satisfaction indicator*" OR "family satisfaction instrument*" OR "family satisfaction inventor*" OR "family satisfaction measur*" OR "family satisfaction outcome*" OR "family satisfaction questionnaire*" OR "family satisfaction scale*" OR "family satisfaction survey*" OR "Health care outcome* assess*" OR "Health care outcome* index*" OR "Health care outcome* indicator*" OR "Health care outcome *instrument*" OR "Health care outcome* inventor*" OR "Health care outcome* measur*" OR "Health care outcome* questionnaire*" OR "Health care outcome* scale*" OR "Health care outcome* screen*" OR "Healthcare outcome* survey*" OR "Healthcare outcome* assess*" OR "Healthcare outcome* index*" OR "Healthcare outcome* indicator*" OR "Healthcare outcome* instrument*" OR "Healthcare outcome* inventor*" OR "Healthcare outcome* measur*" OR "Healthcare outcome* questionnaire*" OR "Healthcare outcome* scale*" OR "Healthcare outcome* screen*" OR "Healthcare outcome* survey*" OR "Health outcome assess*" OR "Health outcome index*" OR "Health outcome indicator*" OR "Health outcome instrument*" OR "Health outcome inventor*" OR "Health outcome measur*" OR "Health outcome questionnaire*" OR "Health outcome scale*" OR "Health outcome screen*" OR "Health outcome survey*" OR "health status assess*" OR "Health status index*" OR "Health status indicator*" OR "health status instrument*" OR "Health status inventor*" OR "Health status measur*" OR "Health status outcome*" OR "Health status questionnaire*" OR "Health status scale*" OR "Health status screen*" OR "Health status survey*" OR "Patient-centred experience" OR "Patient-centered experience" OR "Patient-centred outcome*" OR "Patient-centered outcome*" OR "Patient experience assess*" OR "Patient experience index*" OR "Patient experience indicator*" OR "Patient experience instrument*" OR "Patient experience measur*" OR "Patient experience outcome*" OR "Patient experience questionnaire*" OR "Patient experience screen*" OR "Patient experience scale*" OR "Patient experience survey*" OR "Patient outcome* assess*" OR "Patient outcome* index*" OR "Patient outcome* indicator*" OR "Patient outcome* instrument*" OR "Patient outcome* inventor*" OR "Patient outcome* measur*" OR "Patient outcome* questionnaire*" OR "Patient outcome* screen*" OR "Patient outcome* scale*" OR "Patient outcome* survey*" OR "Patient-orientated assess*" OR "Patient-orientated index*" OR "Patient-orientated indicator*" OR "Patient-orientated instrument*" OR "Patient-orientated measur*" OR "Patient-orientated questionnaire*" OR "Patient-orientated scale*" OR "Patient-orientated survey*" OR "Patient-oriented assess*" OR "Patient-oriented index*" OR "Patient-oriented indicator*" OR "Patient-oriented instrument*" OR "Patient-oriented inventor*" OR "Patient-oriented measure*" OR "Patient-oriented questionnaire*" OR "Patient-oriented scale*" OR "Patient-oriented screen*" OR "Patient-oriented survey*" OR "patient-reported experience*" OR "patient-reported outcome*" OR "patient-reported satisfaction*" OR "patient satisfaction assess*" OR "patient satisfaction index*" OR "patient satisfaction indicator*" OR "patient satisfaction instrument*" OR "patient satisfaction inventor*" OR "patient satisfaction measur*" OR "patient satisfaction outcome*" OR "patient satisfaction questionnaire*" OR "patient satisfaction scale*" OR "patient satisfaction screen*" OR "patient satisfaction survey*" OR "People-centred assess*" OR "People-centred index*" OR "People-centred instrument*" OR "People-centred measur*" OR "People-centred outcome*" OR "People-centred screen*" OR "People-centred scale*" OR "People-centred survey*" OR "patient-centred healthcare outcomes" OR "patient-centred health care outcomes" OR "patient-centered healthcare outcomes" OR "patient-centered health care outcomes" OR "People-centered assess*" OR "People-centered index*" OR "People-centered indicator*" OR "People-centered instrument*" OR "People-centered measur*" OR "People-centered outcome*" OR "People-centered screen*" OR "Person-centered outcome*" OR "Person-centred outcome*" OR "person-reported outcome*" OR "person-reported experience*" OR "PREM" OR "PREMs" OR "PROM" OR "PROMs" OR "PROMIS" |
